# Emergence of gut colonization of carbapenem resistant Gram negatives in intensive care unit patients: a report from a middle income country

**DOI:** 10.1186/2047-2994-4-S1-P135

**Published:** 2015-06-16

**Authors:** E Alp, D Altun, E Berk, A UluKilic, A Ulgey, R Coskun, H Kilic

**Affiliations:** 1 Erciyes University, Kayseri, Turkey

## Introduction

Carbapenem resistant gram negatives (CRGN) have emerged in intensive care units (ICUs) worldwide.

## Objectives

The aim of this study was to identify the incidence of gut colonization by CRGN, risk factors, evaluation of colonized patients for nosocomial infection.

## Methods

Patients (>15 years) admitted to Medical and Anesthesiology ICUs and stayed >48 hours were screened for rectal colonization by CRGN for eighth months. Rectal swab was obtained from each patient weekly and swabbed on selective CHROMagar TMKPC. Any bacterial colony isolated from the selective media was Gram stained and then species identification was performed by API20E/20NE. Patients colonized on admission were excluded. Study population was followed up for CRGN infection during their stay.

## Results

During the study period, 945 patients admitted to ICUs; however 419 patients stayed less than 48 hours and 195 patients were colonized by CRGN on admission. 331 patients enrolled into the study. The median age was 66 (16-98) and 50.5% was female. Rectal colonization was detected in 95 (29%) patients and 150 CRGN bacteria were identified. These microorganisms were; *Acinetobacter baumannii* (73-49%), *Pseudomonas aeruginosa* (32-21%), *Klebsiella pneumoniae* (26-17%), *Enterobacter cloacae* (6-4%) and other CRGN (13-9%).The median time for colonization was 11 (3-55) days. In multivariate analysis, the most significant risk factors for colonization were; length of stay in ICUs, respiratory failure, intubation, use of steroids, transfusion, central venous catheterization and use of colistin. Forty seven nosocomial infection episode with CRGN were detected in 36 (38%) colonized patients and the same pathogen was detected in 37 (78%) episodes. In 24 (66.7%) patients, colonization was detected before the onset of nosocomial infection. On the other hand, seven nosocomial infection episode with CRGN was detected in 5 (2%) non-colonized patient (p<0.05).

## Conclusion

Colonization with CRGN bacteria is an emerging problem in ICUs and is a significant risk factor for the development of nosocomial infections. Rectal screening of high risk ICU patients is needed for infection control and appropriate consideration of empirical treatment in severe ICU patients.

## Disclosure of interest

None declared.

